# Thelazia callipaeda infection during phacoemulsification cataract surgery: a case report

**DOI:** 10.1186/s12886-021-02117-9

**Published:** 2021-10-23

**Authors:** Yang Li, Jie Liu, Qingmei Tian, Dadong Guo, Dongmei Liu, Xianzhen Ma, Hongsheng Bi

**Affiliations:** 1 Shandong University of Traditional Chinese Medicine, No. 16369#, Jingshi Road, Jinan, 250014 People’s Republic of China; 2 Affiliated Eye Hospital of Shandong University of Traditional Chinese Medicine, No. 48#, Yingxiongshan Road, Jinan, 250002 People’s Republic of China; 3 Shandong Provincial Key Laboratory of Integrated Traditional Chinese and Western Medicine for Prevention and Therapy of Ocular Diseases, No. 48#, Yingxiongshan Road, Jinan, 250002 People’s Republic of China; 4 Key Laboratory of Integrated Traditional Chinese and Western Medicine for Prevention and Therapy of Ocular Diseases in Universities of Shandong, No. 48#, Yingxiongshan Road, Jinan, 250002 People’s Republic of China; 5 Eye Institute of Shandong University of Traditional Chinese Medicine, No. 48#, Yingxiongshan Road, Jinan, 250002 People’s Republic of China

**Keywords:** Case report, Thelazia callipaeda, Infection, Phacoemulsification cataract surgery

## Abstract

**Background:**

In August 2020, we found one case of thelazia callipaeda infection during phacoemulsification cataract surgery. This maybe the first report for thelazia callipaeda discovered during phacoemulsification cataract surgery in China.

**Case presentation:**

An 85 years old farmer was found thelazia callipaeda infection during phacoemulsification cataract surgery. The patient admitted whose foreign body sensation was often found in the right eye in recent 2 months. The worm was then taken out with ophthalmic forceps and put into sterile normal saline. The worm was sent to the Eye Institute of Shandong University of Traditional Chinese Medicine for identification. After identification, the worm was regarded as the male thelazia callipaeda. The head is blunt and round, the tail end curls to the abdomen, and the long copulation spines protrudes from the cloaca. The conjunctival sac was washed carefully with a large amount of Sodium Lactate Ringer ‘s Solution. After operation, antibiotics, pranoprofen eye drops, and tobramycin dexamethasone eye drops were further applied. After continuous examination of conjunctival sac for 2 weeks, the patient’s visual acuity maintained 20/20, confirming that there was no residual thelazia.

**Conclusions:**

This report highlights the physician should ask the patient’s history carefully before operation and it is necessary to strengthen health publicity and education, maintaining clean environment and keeping personal eye hygiene.

## Background

Thelazia callipaeda is a zoonotic parasite transmitted by Drosophila, mainly distributed in South Asia, Russia and China. The main vector of the parasite is Okada’s eye-drosophila. Therefore, the prevalence of thelaziasis is also related to the breeding season of *Drosophila melanogaster* (between June and September) [1]. In August 2020, we found one case of thelazia callipaeda infection during phacoemulsification cataract surgery. The details of the diagnosis and treatment of this case are as follows.

## Case presentation

**Fig. 1:**
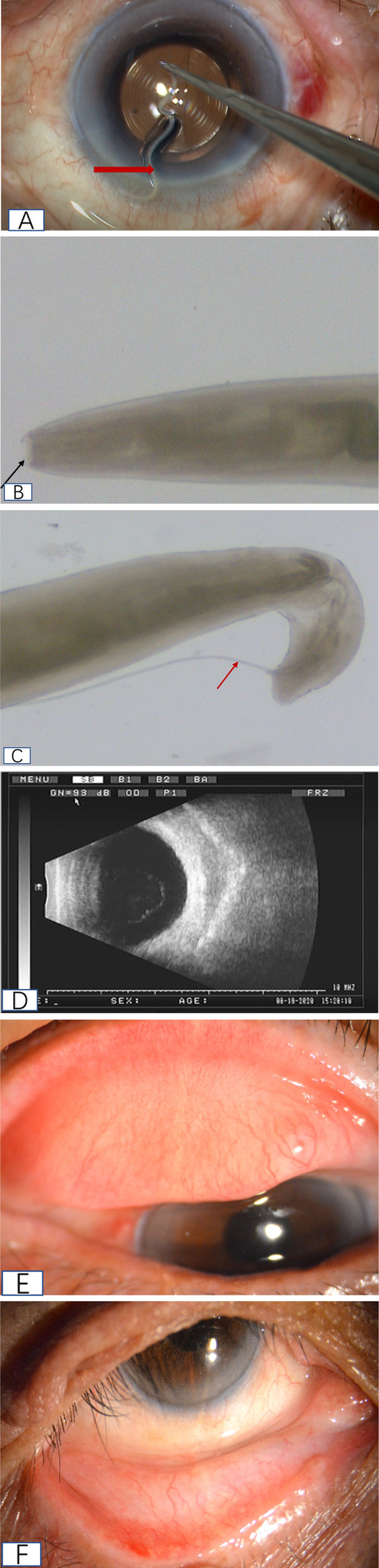
(**a**)Video capture of the operation: during the operation, thelazia conjunctiva (thick red arrow) was found in the conjunctival sac of the upper eyelid (**b**) a blunt rounded head (black arrow) is shown under a microscope (**c**) microscopically showing the long copulation spines of the insect body (fine red arrow) (**d**) B-ultrasound showed that the vitreous body of the right eye was turbid without abnormal strong echo (**e**) conjunctival congestion and follicular formation in the upper eyelid of the patient’s right eye (**f**) mild conjunctival congestion congestion in the lower eyelid of the patient’s right eye

An 85 years old farmer, male, came to the Affiliated Eye Hospital of Shandong University of Traditional Chinese Medicine on August 8, 2020 due to vision loss in the right eye for one year. This patient was admitted to be the inpatient as “age-related cataract”, and then the routine preoperative examination, antibiotic eye drops and pranoprofen eye drops were employed. Two days later, cataract phacoemulsification surgery combined with intraocular lens implantation for cataract was performed. The lacrimal passage was washed routinely prior to the operation, and 0.5% povidone-iodine and Sodium Lactate Ringer’s Solution were used to flush the conjunctival sac during the operation. The phacoemulsification cataract surgery was performed successfully. When the incision was watertight, it was found that the worm moved out from the conjunctival sac of the upper eyelid (as shown in Fig. 1a and video). The worm was milky white, translucent, slender and cylindrical, with a length of approximate 12 mm. The worm was then taken out with ophthalmic forceps and put into sterile normal saline. The upper and lower conjunctival sacs were carefully examined under the operating microscope, and no worm was found. The conjunctival sac was washed carefully with a large amount of Sodium Lactate Ringer’s Solution. Tobramycin and dexamethasone eye ointment were used to cover the eyes and return to the ward. The worm was sent to the Eye Institute of Shandong University of Traditional Chinese Medicine for identification. After identification, the worm was regarded as the male thelazia callipaeda. The head is blunt and round, the tail end curls to the abdomen, and the long copulation spines protrudes from the cloaca (Figs. 1b and c). After questioning the patient’s medical history, the patient admitted whose foreign body sensation was often found in the right eye in recent 2 months, but no medical treatment. There was no obvious foreign body sensation in the left eye, and no history of breeding animals occurred on him.

After operation, antibiotics eye drops, pranoprofen eye drops, and tobramycin dexamethasone eye drops 4 times per day were further applied. On day 1 after operation, the visual acuity was 20/20, intraocular pressure was 12 mmHg, conjunctival hyperemia, cornea was transparent, anterior chamber was moderate depth, atrial flash was (+), and IOL was in normal position. For 5 consecutive days, the conjunctival sac and anterior segment of the operated eyes were carefully examined under the slit lamp, and no abnormality was found. B ultrasound examination showed that the vitreous bodies of both eyes were turbid, and no abnormal strong echo was found (Fig. 1d). The foreign body sensation disappeared after the operation. One week later after the operation, the patient was discharged and reexamined one week later. The result indicated that no thelazia was found in conjunctival sac of both upper and lower eyelids (as shown in Figs. 1e and f).

## Discussion and conclusions

Thelazia conjunctiva is mainly parasitic in the eyes of mammals including cats, dogs, and human eyes. It is common to infect monocular conjunctival sac, and can also parasitize in lacrimal duct, anterior chamber and vitreous body. At the early stage of the disease, there were no obvious symptoms, and the later manifestations of the eye were foreign body sensation, increased secretion and tears. In this case, conjunctival congestion and follicular formation in the upper eyelid of the right eye were considered to be related to the mechanical stimulation of the worm and the chemical stimulation of the excreta. The prevalence of domestic dogs and the widespread distribution of Drosophila, together with unclean eye hygiene, are the main factors for the prevalence of conjunctival sucking nematode disease [1]. In this study, the patient was from a rural area of Shandong Province, China. There was no history of breeding animals in recent years, and the onset time was in the peak period of Drosophila breeding. Thus, it was speculated that the infection was related to Drosophila.

The patient was an older man, complained of blurred vision, did not mention foreign body sensation, and meibomian gland dysfunction in both eyes, which may be the main reason why the resident failed to examine the conjunctival sac carefully. In addition, the hearing loss of the patient and the use of dialects in daily communication increased the difficulty of doctor-patient communication. Therefore, the physician-in-charge could not find any abnormality before operation though thelazia callipaeda could be found after the upper eyelid was carefully opened. Moreover, no abnormalities were found because the eyelid was covered by the blepharoscope during surgery. Thus, we did not perform the upper eyelid examination routinely, and this also provides an important lesson for us. After the thelazia was found during the operation, we immediately took remedial measures, carefully examined the conjunctival sac of both eyes and washed it strictly. The patient received anti-inflammatory drugs after operation. It was reported that thelazia also existed in vitreous cavity [2–5], so, we further examined whether thelazia existed in the vitreous cavity by B ultrasound. After continuous examination of conjunctival sac for 2 weeks, the patient’s visual acuity maintained 20/20, confirming that there was no residual thelazia. During the follow-up, the patient’s family members were told that the patient needed to come back for a follow-up visit one month later. If the patient had foreign body sensation, increased secretion or vision loss, he should come to the clinic immediately at any time, and the doctor-in-charge should be responsible for the follow-up.

To our knowledge, this maybe the first report for thelazia callipaeda discovered during phacoemulsification cataract surgery in China. The case prompts that the physician should ask the patient’s history carefully before operation. When the patient has difficulty in communication, we should fully understand the patient’s symptoms through their family members, so as to avoid missed diagnosis. If thelazia is found during the operation, the worm and eggs should be removed completely to avoid residual and the anti-inflammatory drugs should be used. Even if there is an incision, the prognosis would be better. In addition, the effective approach to prevent and control thelaziasis conjunctival is to actively eliminate the main sources of infection and media, especially in the peak period of Drosophila breeding. Overall, it is necessary to strengthen health publicity and education, maintaining clean environment and keeping personal eye hygiene.

In summary, a detailed history and careful slit-lamp examination are the most important before cataract surgery. Prior to surgery, it should be more careful to do eye examination, including upper eyelid conjunctival sac. As confirmed in our case report, patients with surgical incisions can have a good prognosis after strict irrigation of the onjunctival sac to confirm the absence of worms. After careful verification of the absence of worms, vermifuge or special medical treatments are not necessary.

The patient and his family were satisfied with the treatment. They said the doctor found the thelazia through careful examination, gave antibiotics eye drops, pranoprofen eye drops, and tobramycin dexamethasone eye drops to the eyes to prevent the infection, and ordered a B-ultrasound examination to rule out the possibility of vitreous bodies infection. And he said to continue to keep eyes clean later.

## Data Availability

Data sharing is not applicable to this article as no datasets were generated or analysed during the current study.

## Abbreviation

IOL: intraocular lens
